# Application of Rigidity Theory to the Thermostabilization of Lipase A from *Bacillus subtilis*

**DOI:** 10.1371/journal.pcbi.1004754

**Published:** 2016-03-22

**Authors:** Prakash Chandra Rathi, Alexander Fulton, Karl-Erich Jaeger, Holger Gohlke

**Affiliations:** 1 Institute for Pharmaceutical and Medicinal Chemistry, Department of Mathematics and Natural Sciences, Heinrich-Heine-University, Düsseldorf, Germany; 2 Institute of Molecular Enzyme Technology, Heinrich-Heine-University, Düsseldorf, Germany; 3 Institute of Bio- and Geosciences IBG-1: Biotechnology, Forschungszentrum Jülich GmbH, Jülich, Germany; Weizmann Institute of Science, ISRAEL

## Abstract

Protein thermostability is a crucial factor for biotechnological enzyme applications. Protein engineering studies aimed at improving thermostability have successfully applied both directed evolution and rational design. However, for rational approaches, the major challenge remains the prediction of mutation sites and optimal amino acid substitutions. Recently, we showed that such mutation sites can be identified as structural weak spots by rigidity theory-based thermal unfolding simulations of proteins. Here, we describe and validate a unique, ensemble-based, yet highly efficient strategy to predict optimal amino acid substitutions at structural weak spots for improving a protein’s thermostability. For this, we exploit the fact that in the majority of cases an increased structural rigidity of the folded state has been found as the cause for thermostability. When applied prospectively to lipase A from *Bacillus subtilis*, we achieved both a high success rate (25% over all experimentally tested mutations, which raises to 60% if small-to-large residue mutations and mutations in the active site are excluded) in predicting significantly thermostabilized lipase variants and a remarkably large increase in those variants’ thermostability (up to 6.6°C) based on single amino acid mutations. When considering negative controls in addition and evaluating the performance of our approach as a binary classifier, the accuracy is 63% and increases to 83% if small-to-large residue mutations and mutations in the active site are excluded. The gain in precision (predictive value for increased thermostability) over random classification is 1.6-fold (2.4-fold). Furthermore, an increase in thermostability predicted by our approach significantly points to increased experimental thermostability (*p* < 0.05). These results suggest that our strategy is a valuable complement to existing methods for rational protein design aimed at improving thermostability.

## Author Summary

Protein thermostability is a crucial factor for biotechnological enzyme applications. However, performance studies of computational approaches for predicting effects of mutations on protein (thermo)stability have suggested that there is still room for improvement. We describe and validate a novel and unique strategy to predict optimal amino acid substitutions at structural weak spots. At variance with other rational approaches, we exploit the fact that in the majority of cases an increased structural rigidity of the folded state is the underlying cause for thermostability. When applied prospectively on lipase LipA from *Bacillus subtilis*, a high success rate in predicting thermostabilized lipase variants and a remarkably large increase in their thermostability is achieved. This demonstrates the value of the novel strategy, which extends the existing portfolio of methods for rational protein design.

## Introduction

Thermostability is a crucial factor for a wealth of biotechnological enzyme applications [1,2]. Protein engineering aimed at improving thermostability is thus an important field of research in biotechnology [3,4]. There, methods of directed evolution are usually applied, which mimic natural evolution [5–8]. However, directed evolution is limited in that out of the extraordinarily large number of possible variant proteins, only a small subset can be experimentally tested [9]. Alternatively, rational approaches have been successfully pursued [10–13] but the major challenge here remains the prediction of mutation sites and the optimal amino acid substitution at such sites [14,15].

**Fig 1:**
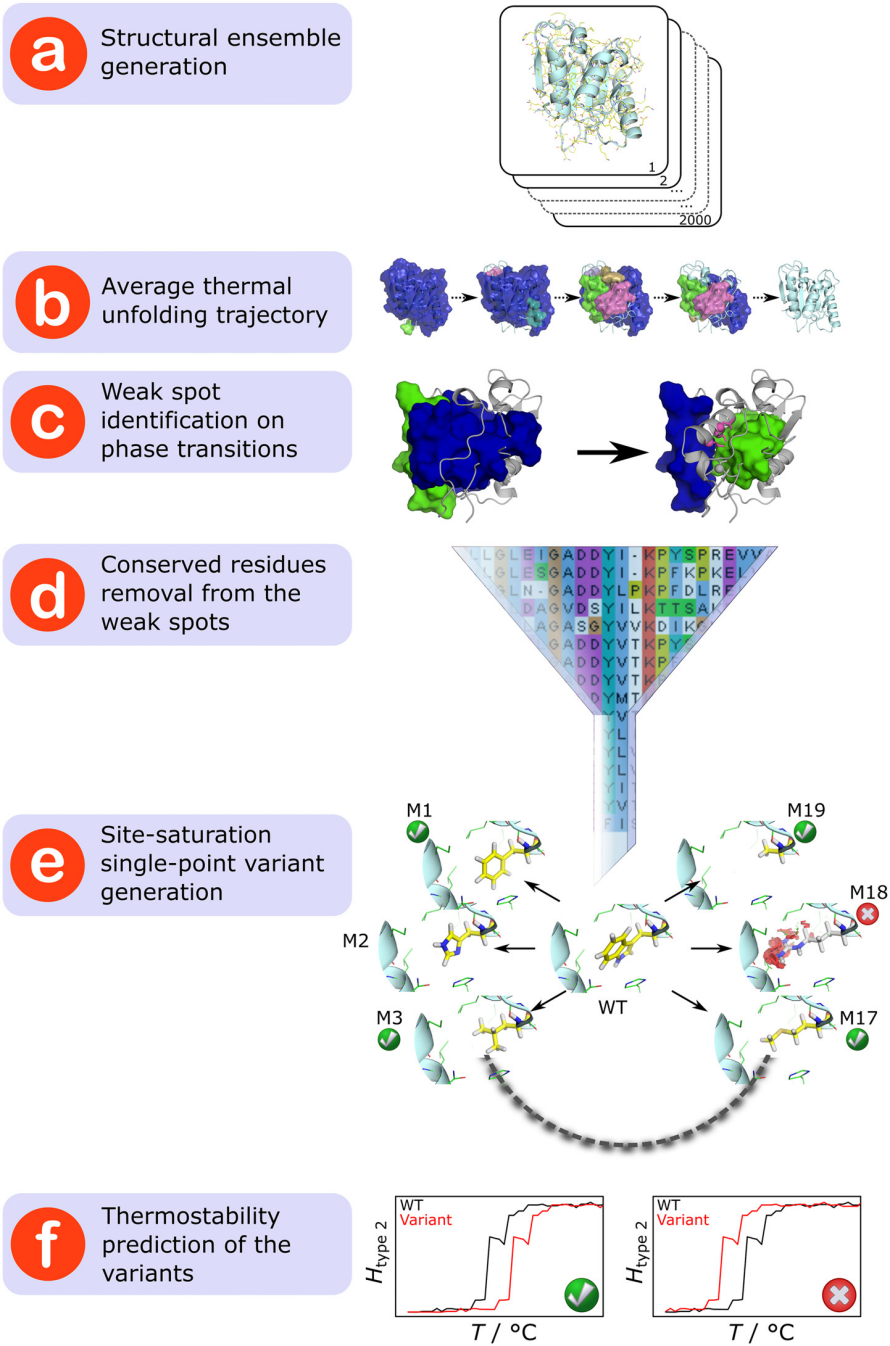
Strategy to rationally predict mutations that increase structural rigidity and thermostability. (a) A structural ensemble of the respective protein is generated by MD simulations. (b) The average thermal unfolding trajectory depicting a decomposition into rigid clusters (in the order of decreasing size colored in blue, green, magenta, cyan, orange, and violet) for each step of the unfolding simulation is created by subjecting the structural ensemble to CNA. (c) For every major transition during the thermal unfolding, weak spot residues (depicted as a sphere for the C_α_ atom and sticks for the side-chain) are identified. (d) Weak spot residues that are highly conserved across the protein family (≥ 80% identity) are removed from the weak spot list. (e) For the rest, structures of site-saturation single-point variants (termed M1–M19) are generated. Mutations that lead to energetically unfavorable structures (indicated by red discs around the mutated residue in the case of M18) are discarded. (f) For each variant, the phase transition temperature *T*_p_ is computed using CNA; a higher *T*_p_ value than that of the WT protein indicates a thermostabilizing mutation.

As to the prediction of mutation sites, we developed the rigidity theory-based Constraint Network Analysis (CNA) approach [16–21] (available as a web service at http://cpclab.uni-duesseldorf.de/cna/ [16–21]), which identifies residues in a protein that are structural “weak spots”. For this, a protein is modeled as a network of sites (atoms) and constraints (covalent and noncovalent interactions) [22]. Rigid atom clusters and flexible regions in between are then rigorously determined by rigidity analysis [23–25]. By successively removing non-covalent constraints from the network, the thermal unfolding of the protein is simulated (Fig 1a and 1b) [16,18,19,26]. From the unfolding trajectory, a phase transition temperature *T*_p_ is identified, which relates to the (thermodynamic) thermostability, as are the weak spots (Fig 1c). Mutating such weak spots should likely improve a protein’s thermostability [16,18,19].

Here, we describe and validate a novel and unique strategy based on the CNA approach to predict optimal amino acid substitutions at these weak spots. At variance with other rational approaches that rely upon calculating free energies for predicting effects of mutations on a protein’s thermostability [27–33], we exploit the fact that in the majority of cases an increased structural rigidity of the folded state has been identified as the underlying cause for thermostability [34]. To this end, we add a highly efficient, ensemble-based second step by generating structural models of single-point site-saturation mutations at identified weak spots, filtering the models with respect to their structural quality, and screening for variants with increased structural rigidity (Fig 1d–1f, see below for detailed descriptions). Using the recently developed ENT^FNC^ approach [35] that performs rigidity analyses on an *e*nsemble of *n*etwork *t*opologies generated from a single input structure using *f*uzzy *n*etwork *c*onstraints, rather than a structural ensemble, this second step only takes about 1 h on a single core per variant and can be performed in parallel for multiple variants. We applied this strategy prospectively on lipase LipA from *Bacillus subtilis* (*Bs*LipA); *Bs*LipA has considerable biotechnological importance [36,37] and has been extensively studied with respect to thermostability [6,15,38–43], which makes *Bs*LipA a prominent model system. Out of 589 *Bs*LipA variants screened *in silico*, twelve were suggested for experimental testing. Of these, three showed a significant increase of up to 6.6°C in thermostability with respect to the wild-type enzyme (WT). We thus achieved both a high success rate in predicting thermostabilized lipase variants and a remarkably large increase in the thermostability of such variants. This demonstrates the value of the novel strategy, which extends the existing portfolio of methods for rational protein design aimed at improving thermostability.

## Results

### Predicting thermostabilizing mutations

**Fig 2:**
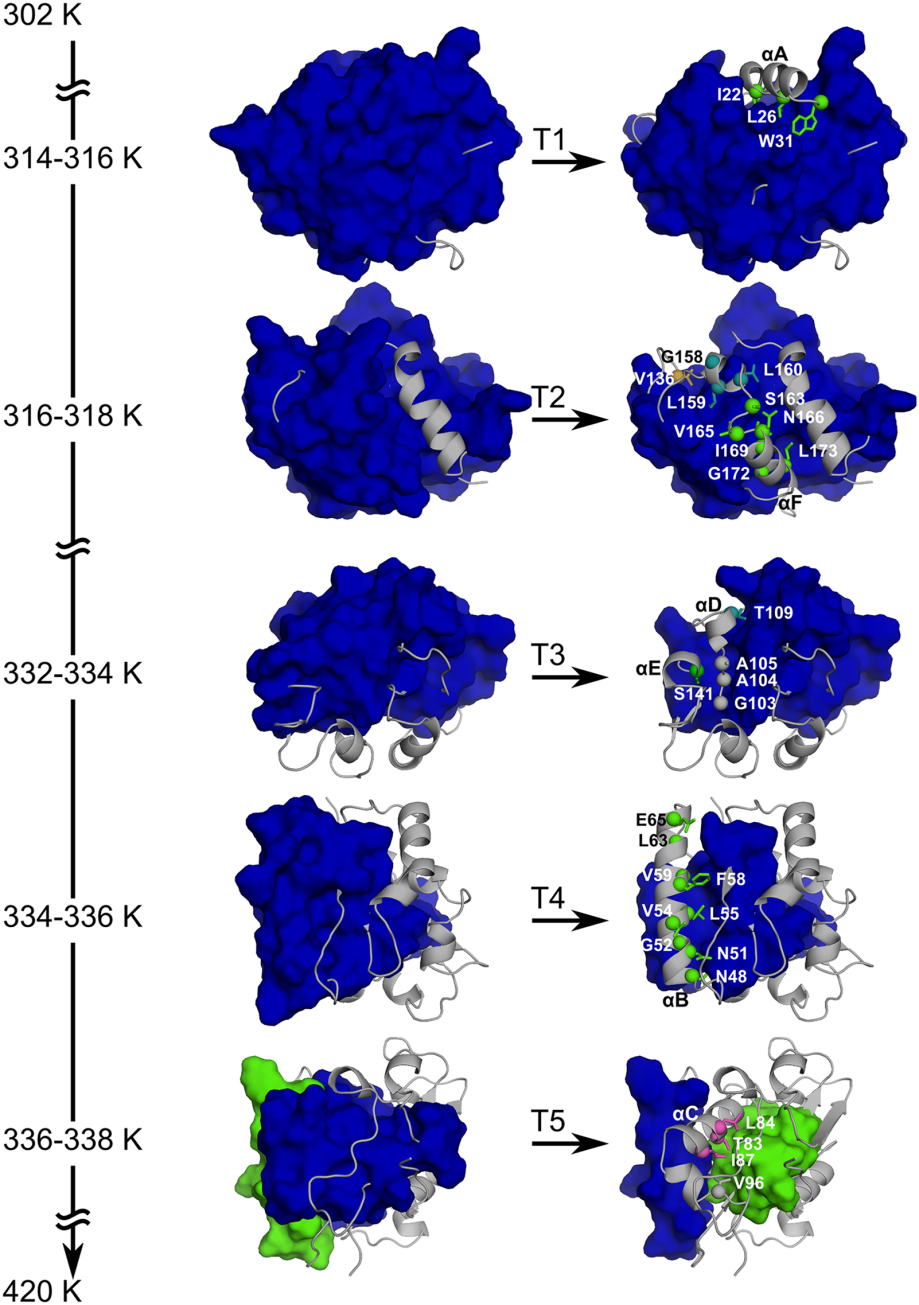
Thermal unfolding trajectory of WT *Bs*LipA showing transitions for which weak spot residues were identified. Uniformly colored bodies represent rigid clusters; for clarity, only the largest rigid cluster (blue) is shown for the first four transitions (T1–T4), and the two largest rigid clusters (blue and green) are shown for the last transition (T5). C_α_ atoms of the identified weak spot residues are shown as spheres, and side-chain atoms are shown in stick representation. Weak spot residues are colored according to the rigid cluster they are part of (rigid clusters are assigned blue, green, magenta, cyan, and orange colors in the descending order of their size in terms of the number of residues); a weak spot residue colored in gray indicates that it is part of a rigid cluster composed of less than three residues. Weak spot residues that are highly conserved in the multiple sequence alignment of the lipase family are not shown. Important helices that segregate from the largest rigid cluster at the respective transition are labeled.

*Bs*LipA has a minimal α/β hydrolase fold in which a central parallel β-sheet of six β-strands is surrounded by six α-helices [44]. For identifying weak spots on *Bs*LipA, a thermal unfolding simulation was carried out by CNA on an ensemble of 2000 WT *Bs*LipA structures extracted from a molecular dynamics (MD) trajectory of 100 ns length (Fig 1a). The ensemble-based CNA was pursued to increase the robustness of the rigidity analyses [19,35,45]. The unfolding trajectory (Figs 1b and 2) reveals the early segregation of loops from the largest rigid cluster, followed by the segregation of α-helices and, finally, the segregation and disintegration of the β-sheet region. This order of segregation is in agreement with experimental findings on the unfolding of other α/β hydrolase proteins [46,47]. The realistic description of WT *Bs*LipA thermal unfolding encouraged us to identify weak spots at major phase transitions along the unfolding trajectory (Fig 1c). By visual inspection of the unfolding trajectory, we identified five major transitions (T1–T5) at which helices αA, αF, αD and αE, αB, αC as well as the central beta sheet segregate from the largest rigid cluster at temperatures 316, 318, 334, 336, and 338 K, respectively (Table 1 and Fig 2).

**Table 1:** Phase transition points at which weak spot residues are identified during the thermal unfolding simulation of *Bs*LipA.

Phase transition	Temperature of the phase transition^[a]^	Major secondary structures segregating from the giant rigid cluster	Weak spot residues^[b]^
T1	314–316	αA	I22, L26, W31
T2	316–318	αF	**D133**, V136, G158, L159, L160, S163, V165, N166, I169, G172, L173
T3	332–334	αD and αE	G103, G104, A105, **N106**, T109, S141
T4	334–336	αB	N48, N51, G52, V54, L55, F58, V59, **V62**, L63, E65, **T66**, **V71**
T5	336–338	αC and central β sheet	T83, L84, I87, V96

^[a]^ In K.

^[b]^ Residues in bold are highly conserved in the multiple sequence alignment; see the main text for details.

Weak spot residues were then identified as those residues that are in the neighborhood of the largest rigid cluster from which they segregate at the respective major transition. These residues are particularly promising for increasing *Bs*LipA’s thermostability considering that their mutation can improve the interaction strength with the largest rigid cluster and, hence, delay the disintegration of that cluster with increasing temperature. In total, 36 weak spots were found, which are located on α-helices and loops joining α-helices and β-strands (Fig 2). The weak spot residues are very diverse in size (ranging from Gly to Trp) and physicochemical properties (charged, uncharged polar, and hydrophobic) (Table 1). Of these, weak spot residues at highly conserved sequence positions were discarded (Figs 1d and S1; Table 1) because conserved residues are usually important for function and/or stability of a protein and, hence, should not be mutated [48,49].

For each of the remaining 31 weak spots (~17% of all *Bs*LipA residues), computational site saturation mutagenesis was performed by generating structures of all possible single-point amino acid substitutions using the SCWRL program (Fig 1e) [50]. SCWRL constructs variant models by predicting backbone-dependent side-chain conformations with the help of a rotamer library. This resulted in 589 single point variants. 67 variant structures were discarded based on the evaluation of residue-wise non-local interaction energies by the ANOLEA server (S1 Fig) [51,52]. In such structures, the mutation apparently does not fit into the environment of the other residues.

The remaining 522 variants were subjected to thermal unfolding simulations on ensembles of network topologies using the ENT^FNC^ approach [35] implemented in CNA. Differences in the phase transition temperatures Δ*T*_p_ = *T*_p_ (variant) − *T*_p_ (WT) were averaged over 1000 simulations started from different network topologies generated for each variant (see “Materials and Methods section”; Fig 1f). A map of Δ*T*_p_ values of all variants is shown in S1 Fig. In total, this procedure yielded a predicted thermostabilization with respect to WT *Bs*LipA for 75 out of the 522 mutations (~14%) investigated. In order to further reduce the number of mutations for experimental validation only the mutation with the highest Δ*T*_p_ was chosen from all mutations with Δ*T*_p_ > 1 K at a weak spot. The sole exception is G104 located in the active site, for which two mutations were chosen. This resulted in twelve lipase variants of which the most are associated with weak spot residues on helix αB identified during the late transition T4 (Table 2; S1 Fig).

**Table 2:** *Bs*LipA variants with positive Δ*T*_p_ characterized experimentally.

*Bs*LipA variant^[a]^	Location of the mutation on secondary structure element	Phase transition of weak spot identification	Predicted Δ*T*_p_^[b]^	*T*′_50_^[c]^
Wild-type	-	-	-	49.10
I22W	αA	T1	2.80	44.89
N51F	αB	T4	4.30	46.05
G52M	αB	T4	16.47	49.59
**V54H**	αB	T4	2.09	54.80
L55F	αB	T4	3.48	47.62
**F58I**	αB	T4	2.27	55.65
V59F	αB	T4	11.95	49.44
I87W	αC	T5	4.91	-[d]
**V96S**	β6	T5	2.36	52.65
G104I	Loop β6- αD	T3	1.98	-[d]
G104L	Loop β6- αD	T3	5.07	-[d]
L160H	αF	T2	2.25	43.30

^[a]^ Variants highlighted in bold show a significant increase in *T′*_50_ compared to WT.

^[b]^ Difference phase transition temperatures *T*_p_ (variant) − *T*_p_ (WT); in °C.

^[c]^ The temperature at which the fraction of the activity to the initial activity (at 40°C) is 50% after incubating for 30 min; in °C.

^[d]^ No activity after 30 min incubation at temperatures of 40–60°C.

As a negative control, we also predicted 10 variants with negative Δ*T*_p_, i.e., where a mutation according to the thermal unfolding simulations leads to a decrease in thermostability with respect to WT (S1 Table). Six of these mutations were chosen from the above analyses of 522 variants such that they have the most negative Δ*T*_p_; four were chosen with the most negative Δ*T*_p_ from analyses of variants with a mutation not at a weak spot.

### Experimental characterization of thermostability

Initially, specific activities of WT *Bs*LipA and the twelve variants (Table 2) for hydrolysis of *p*-nitrophenyl-palmitate (*p*NPP) were measured at temperatures between 40 and 60°C after keeping them at the respective temperatures for 5 min. WT *Bs*LipA showed the highest specific activity (246 U/mg) among all *Bs*LipA variants at the temperature of maximum activity *T*_max_ (40°C) (S2 Fig). At temperatures above 55°C, the activity begins to drop, which is probably due to an unfolding already within 5 min of preincubation. However, two variants, F58I and V96S, showed higher activities than the WT at temperatures above 58°C (S2 Fig), which may originate from them being more stable at high temperatures.

**Fig 3:**
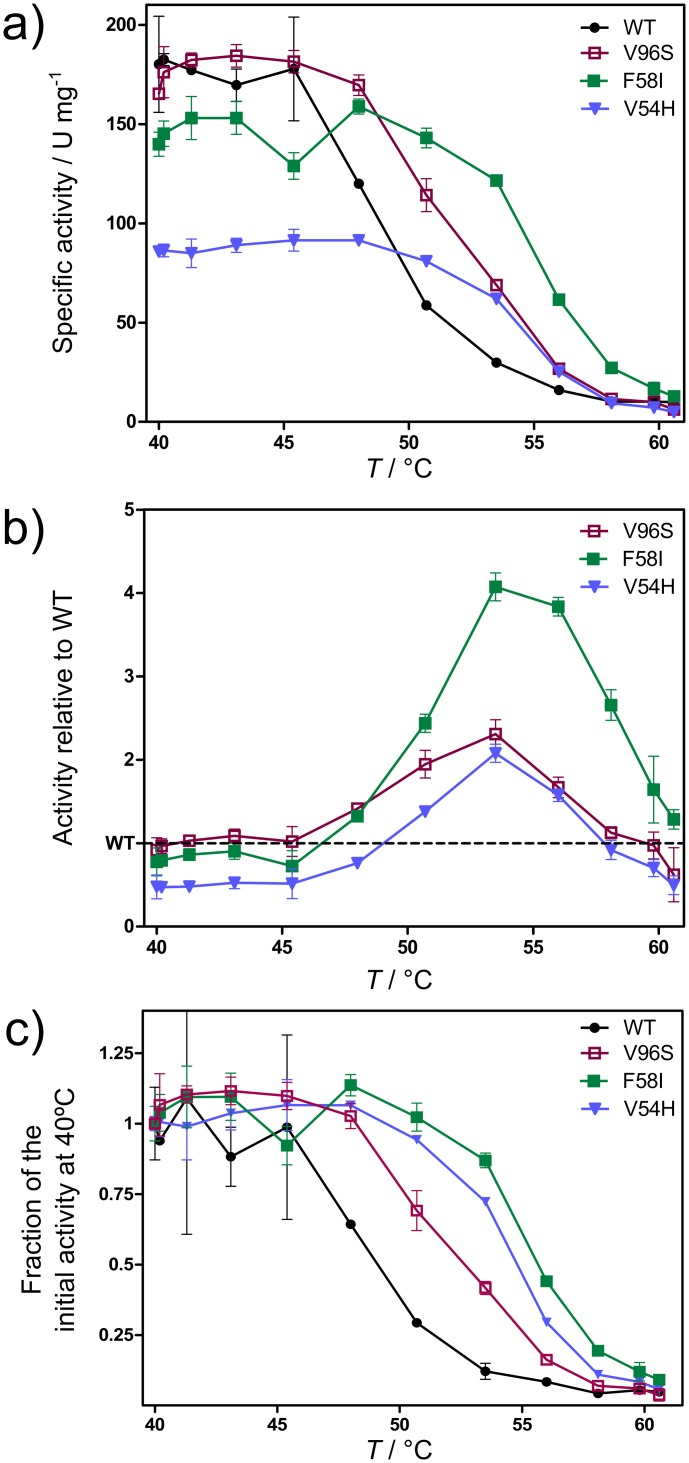
Thermostability of WT (black) and variants V54H (blue), F58I (green), and V96S (red) shown as activity *vs*. temperature curves. The activity was measured at indicated temperatures after incubating for 30 min at these temperatures. Curves show absolute specific activity (a), activity normalized by the activity of WT (b), and the fraction of the activity to the initial activity at 40°C (c).

Next, thermostability was assessed by measuring the activity of each *Bs*LipA variant at temperatures between 40 and 60°C after incubating the respective variant at these temperatures for 30 min. Three variants, V54H, F58I, and V96S, were more thermostable than WT; they consistently showed higher activities than the WT at temperatures above 48°C (Figs 3a and S3). The largest differences between thermostabilities of WT and variants of *Bs*LipA was observed at 53.5°C where the activities of V54H and V96S were twice as a high as that of the WT, and the activity of F58I was four times higher (Fig 3). The kinetic constants of these variants were derived from initial rate measurements for hydrolysis of *p*-nitrophenyl-decanoate (*p*NPD) at 40°C (see S1 Text). No significant impact on the Michaelis constant (*K*_M_) was observed, and the turnover numbers (*k*_cat_) were reduced by at most 25% (Table 3). Thus, the thermostability of the variants has been increased without significantly influencing k_cat_ / K_M_ at 40°C. Still, two of the three thermostable variants showed lower activities than WT at temperatures below ~45°C (Fig 3). This may have been caused by a rigidification of the lipase structure in the thermostable variants (see section “Analysis of thermostability changes at the structural level” below), which may also influence the flexibility of the active site. Similarly, in a series of five orthologs of 2-deoxy-d-ribose-5-phosphate aldolase (DERA) from psychrophilic, mesophilic, and hyperthermophilic organisms investigated by us recently in terms of biochemical, structural, and rigidity properties, an anticorrelation between specific activity at temperatures ≤ 40°C and experimental or computed melting temperature was observed [53]. In that study, both the analysis of local rigidity by CNA and B-factor analysis of X-ray structures provided independent clues that psychrophilic DERAs have a more flexible environment of the substrate binding pocket. Thus, it may depend on the actual operating temperature of an enzymatic process whether it is worth to apply thermostable variants with increased activities at high(er) temperatures only.

**Table 3:** Kinetic parameters of *Bs*LipA variants and wild-type.

Variant	K_M_^[a]^ (μM)	k_cat_ (s^-1^)	k_cat_ / K_M_ (μM^-1^ * s^-1^)
Wild-type	34.72 ± 6.49	926.40 ± 38.81	26.68 ± 6.10
V54H	40.02 ± 8.64	784.60 ± 38.97	19.51 ± 5.18
F58I	36.71 ± 7.83	690.50 ± 33.35	18.80 ± 4.91
V96S	32.30 ± 7.39	785.00 ± 39.73	24.30 ± 6.79

^[a]^ Kinetic parameters were derived from experiments conducted at 40°C using *p*NPD as substrate.

Finally, the thermostability of *Bs*LipA variants was quantified by *T′*_50_ values; these values report on the temperature at which the fraction of the activity to the initial activity (at 40°C) is 50% after incubation for 30 min. This is different from the *T*_50_ values normally used for characterizing the thermostability of proteins [15,54,55] in that the activity here is measured at the temperature of incubation, not at room temperature after cooling. *T′*_50_ thus reports on the thermo-tolerance of an enzyme during operational bioprocesses carried out at elevated temperatures for a longer duration of time, e.g., as done in the lipid processing industry [56]. The three variants V54H, F58I, and V96S showed *T′*_50_ values higher by 5.7, 6.6, and 3.6°C, respectively, than WT *Bs*LipA (Fig 3c; Table 2). The predicted Δ*T*_p_ values for these variants were similar to each other, in agreement with the similar *T′*_50_ values found, but at the lower end of all predicted Δ*T*_p_ (Table 2).

For the variants used as a negative control (S1 Table) [57], the thermostability was quantified by *T′*_50_ values; these values report on the temperature of incubation for 20 min after which the fraction of the activity at room temperature to the initial activity is 50%. With respect to the *T′*_50_ values used above, a significant and very good correlation was obtained for *T′*_50_ (see S1 Text) For nine out of ten variants, significantly lower thermostabilities were measured, with the largest decrease being 7.3°C for the N48R variant (S1 Table).

### Analysis of thermostability changes at the structural level

The three thermostable variants involve mutations at weak spots identified at later phase transitions T4 and T5 during the thermal unfolding simulation. This finding supports our previous reasoning that it is the late phase transition(s) involving the final decay of the rigid core during thermal unfolding that mostly determine(s) the thermodynamic thermostability of a protein [16,18,19]. Accordingly, mutations that strengthen contacts of weak spot residues identified at late phase transitions should particularly improve thermostability. A sound discussion of this implication requires X-ray structural data of the variants, which is not yet available. Still, using the modeled variant structures, we observed that the three variants V54H, F58I, and V96S do have in general stronger “rigid contacts” between neighboring residues than the WT (a “rigid contact” denotes that two residues belong to one rigid cluster): On average, the mutations V54H, F58I, and V96S increased the strength of rigid contacts of neighboring residues by 2.0, 1.2, and 0.4 K, respectively, compared to WT (S4 Fig; see section “Constraint Network Analysis: Local rigidity indices” for an explanation how these values were calculated).

**Fig 4:**
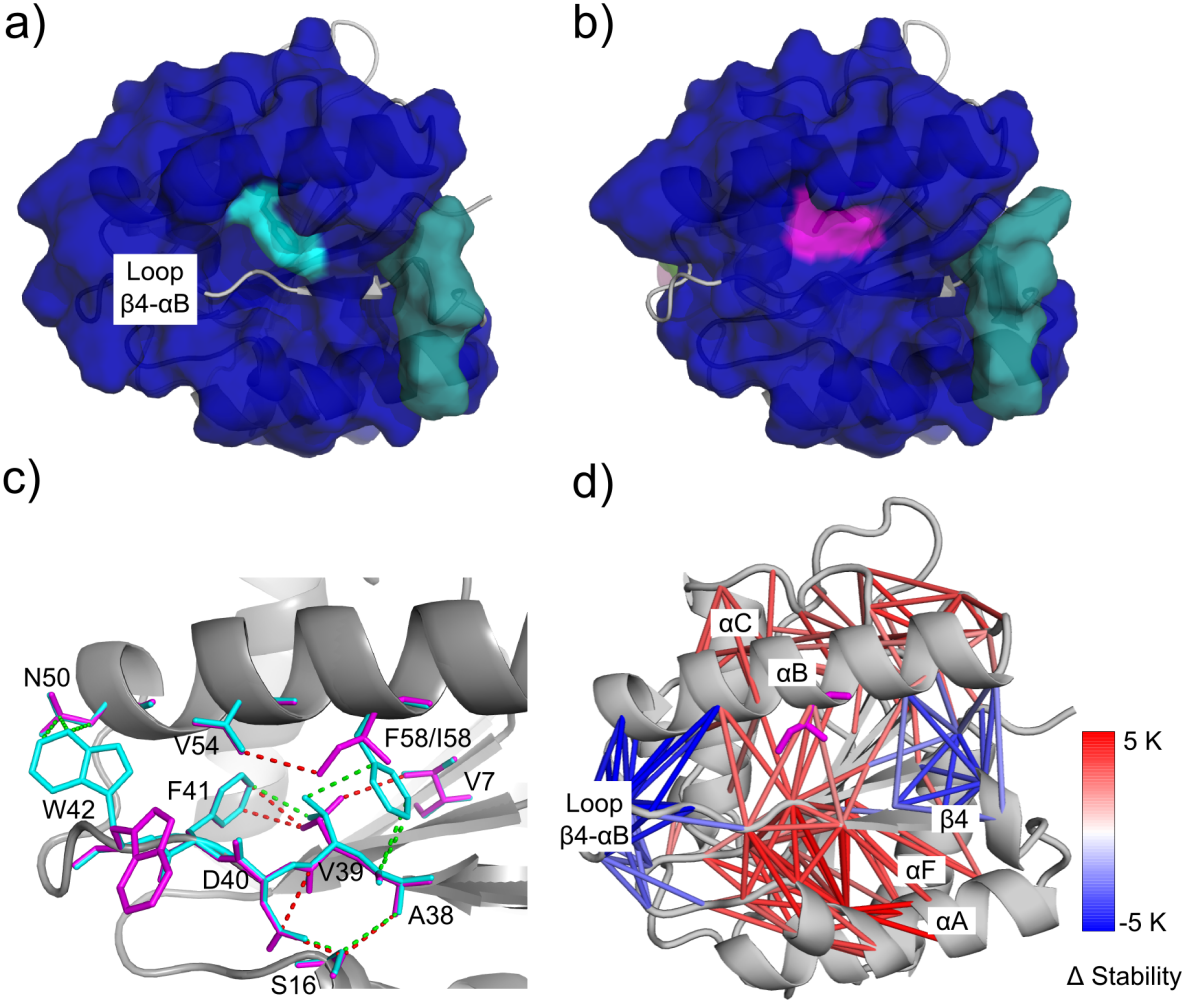
Structural origin of differences in the thermostability of WT and F58I, shown by a rigid cluster decomposition of WT (a) and F58I (b) at 316 K. Rigid clusters are shown as uniformly colored bodies. The mutation site (residue 58), shown by a cyan (a) and a magenta (b) surface, is part of the largest rigid cluster (blue) in both WT and F58I. Hydrophobic contacts in the proximity of the mutation site between carbon atom pairs at most 3.8 Å apart are shown as green (WT) and red (F58I) dashed lines (c). Residues involved in making such contacts are shown as cyan (WT) and magenta (F58I) sticks. Differences in the stability of “rigid contacts” between variant F58I and WT are depicted on the variant structure (d). Two residues form a “rigid contact” if they belong to one rigid cluster. A red (blue) stick connecting C_α_ atoms of two residues indicates that a rigid contact in the variant is more (less) stable than in the WT (see color scale). Only those contacts of variant F58I that are stabilized or destabilized by ≥ 2 K are shown for clarity; for the same reason, contacts between two residues of the same secondary structure element are not shown. The mutated residue I58 is displayed by magenta sticks. Blow-ups of panel d showing the contact stability between secondary structure pairs mentioned in the main text can be found in S5 Fig.

Considering the most thermostable variant F58I in more detail, the strengthening holds true for local contacts as well as contacts that arise from a long-range stabilization. As to local contacts, Ile at position 58 along with residues of the neighboring loop β4-αB (A38, V39, D40) are part of a rigid cluster, which persists to a temperature ~3 K higher than the rigid cluster formed by F58 of WT and the same loop residues (Figs 4a, 4b, S4b and S6a). The persistence at higher temperature results from a better side-chain packing (Fig 4c). In particular, in variant F58I, V39 forms four hydrophobic contacts with three different residues (V7, S16, F41), whereas in WT it only forms two such hydrophobic contacts (Fig 4c). However, not all F58I mutation-induced changes lead to stabilization (Fig 4d). As to contacts that arise from a long-range stabilization, residues of several pairs of secondary structure (αA/β strands 3,4,5; αB/αC; loop αB-β5/loop αC-β6; loop αC-β6/loop αD-β7) remain part of one rigid cluster for temperatures 2–5 K higher in the variant F58I than in WT (Figs 4d, S4b and S5b–S5e). This demonstrates the inherent long-range aspect to rigidity percolation [23,45,58–60], i.e., a local change on one end of a network can affect the stability all across the network.

Recently, we described the unfolding pathway of *Bs*LipA in detail as deduced from thermal unfolding simulations [61] (see also page 9, Fig 3 in that publication). We observed that α-helices αD and αE first segregate to form individual small rigid clusters, followed by αA and αF. The giant rigid cluster at this temperature is formed by the central β-sheet region and the two helices αB and αC. Next, the β-sheet region becomes sequentially flexible, beginning with β4 and β8, followed by the remaining β-strands in the order β3, β7, and β5−β6, finally leading to a completely flexible β-sheet region. As described above, several of these secondary structural elements are involved in the thermal stabilization of the variant F58I (S4 Fig). Furthermore, the amino acids forming the catalytic triad in *Bs*LipA are S77 located between strand β5 and helix αC, D133 between strand β7 and helix αE, and H156 between strand β8 and helix αF [62]. Stabilization of these secondary structural elements due to introducing mutation F58I (S4 Fig; in particular, loops αB-β5 and αC-β6 (S5d Fig), loops αC-β6 and αD-β7 (S5e Fig), and helices αB and αC (S5c Fig)) may thus delay the unfolding of the active site.

Five mutations at weak spots identified at transitions T4 and T5 resulted in lower *T′*_50_ values than that of WT *Bs*LipA (Table 2). This result appears to contradict our reasoning that mutations which strengthen connections of weak spot residues identified at late phase transitions should particularly improve thermostability. In each case, however, a small amino acid was substituted by a large amino acid, which likely could not be accommodated by the fold. This calls for improved modeling considering backbone relaxation [63] for variant construction in future studies with the aim to improve discrimination between amino acid substitutions in already densely packed regions, which could not accommodate small-to-large residue mutations, and substitutions in the vicinity of a protein cavity, where small-to-large residue mutations are an established strategy to increase protein stability [39,64]. Along the same lines, the two variants G104I and G104L out of the three variants that showed a nearly complete loss of activity at room temperature, and no residual activity after 30 min incubation at temperatures between 40–60°C, involved a residue located in the active site. While at the opposite side of the catalytic triad, introducing larger residues may occlude the substrate binding region. Such weak spots can be filtered out in future studies based on their location in the protein [65].

## Discussion

We developed a novel rational approach based on increasing structural rigidity for improving a protein’s thermostability and applied it prospectively to *Bs*LipA. The approach combines ensemble- and rigidity theory-based weak spot prediction by CNA, filtering of weak spots according to sequence conservation, computational site saturation mutagenesis, assessment of variant structures with respect to their structural quality, and screening of the variants for increased structural rigidity by ensemble-based CNA. Two reasons account for its high computational efficiency: In the first step, the number of potential mutation sites is dramatically reduced due to concentrating only on structural weak spots. In the second step, the use of ensembles of network topologies, rather than structural ensembles, alleviates the need for costly conformation sampling. As a result, about one mutation per hour can be processed on one core once weak spots have been detected (Table 4); this task is trivially parallelizable for multiple mutations. From a methodological point of view, this majorly distinguishes our approach from other state-of-the art methods for predicting effects of mutations on protein stability [27–33] in that these methods would need to consider all potential mutation sites due to the lack of an equivalent “step one”. Furthermore, these methods either do not consider ensemble representations of the protein [28–33] or use structural ensembles [27]. Finally, our approach does not require weighting or fitting parameters, in contrast to other methods [27,30,31,66].

**Table 4:** Computing times for weak spot identification, site saturation mutagenesis, and screening for increased structural rigidity.

Step^[a]^	Time required^[b]^	Comment
a) MD simulation	~78 h	100 ns long MD simulation on a single GPU
b) Thermal unfolding simulation	4 h and 35 min	Structural ensemble of 2000 structures run on one CPU core
c) Weak spot detection	2 h	Manual identification by visual inspection
d) Filtering weak spots	Instantaneous	Highly conserved weak spots were discarded
e) Variant modeling by SCWRL	< 1 s	For a single mutation
f) ENT^FNC^ run	~ 1 h and 10 min	For a single mutation applying 1000 network topologies
Total	~700 h	The computations for 522 times step f) (~610 h) can be trivially parallelized.

^[a]^ Steps are according to Fig 1.

^[b]^ For the case study with *Bs*LipA described here. With respect to the protein size *N*, the times required for the steps scale as a) *N* log *N* [86], b) *N* [23], c) *N* (assuming that the number of weak spots scales linearly with the protein size), e) *N*, and f) *N*^2^.

As to the application to *Bs*LipA, our approach resulted in three out of twelve experimentally tested single-point mutations with significantly increased thermostability with respect to WT, yielding 6.6°C as the largest increase. This increase compares favorably to the median increase in the apparent melting temperature of 8°C found for 93 cases of engineered proteins, most of which contain more than one mutation [67]. Considering all tested single-point mutations, our approach yielded a success rate as to significantly increased thermostability of 25%, which raises to 60% if the five small-to-large residue mutations and the two mutations in the active site are excluded. These success rates are markedly higher than the 5% of mutations showing an increase in protein stability found within 1285 variants of ten different proteins [68,69]. It is also instructive to compare our results to those obtained by testing a complete site saturation mutagenesis library of *Bs*LipA for improved detergent tolerance, where the success rate amounts to 2% [57]. Furthermore, for state-of-the-art methods for predicting the sign of stability change due to a mutation, impressive accuracies of over 80% have been reported [28]. These values result from the methods being very good at predicting destabilizing mutations and the prevalence of such mutations in the investigated data sets [28]. In line with this, for our predicted negative controls, we found a success rate as to significantly decreased thermostability of 90%. In contrast, the methods’ performances are much worse in predicting stabilizing mutations, yielding an average success rate for such mutations of 36% over 12 methods [28].

Evaluating the performance of our approach as a binary classifier [70] (S2 Table), our approach discriminates between mutations leading to increased thermostability *versus* those leading to decreased thermostability with a sensitivity of 83%, a specificity of 56%, and an accuracy of 63% considering all variants in Tables 2 and S1, and a sensitivity of 100%, a specificity of 77%, and an accuracy of 83% if the small-to-large residue mutations and the two mutations in the active site are excluded. In our view, this signifies that our approach provides for a robust binary classifier. Our approach has a precision (predictive value for increased thermostability) of 42% (60% if the small-to-large residue mutations and the two mutations in the active site are excluded) (S2 Table), which leads to a gain in precision with respect to a random classifier of a factor of 1.6 (2.4). Furthermore, a Mann–Whitney *U* test [71] demonstrates that predicted positive Δ*T*_p_ significantly points to increased experimental thermostability (*p* < 0.05) (see S2 Text).

An approach related to CNA is the distance constraint model (DCM)[72], which reaches average percent errors of 1.1% (Pearson correlation coefficient *R* = 0.72) [73] and 4.3% (*R* = 0.64) [74] for melting point predictions of protein variants with single and multiple mutations, corresponding to an error of ~4 K [73] and ~14 K [74]. This model requires a system-specific fitting to experimental heat capacity curves from differential scanning calorimetry, however [73,74]. Over all variants predicted (including the negative controls but excluding the three variants for which no activity could be measured (Table 2)), our approach, which does not require fitting parameters, yields a significant (*R* = 0.48, *p* = 0.02) correlation between predicted and experimental thermostabilities; if small-to-large residue mutations and the two mutations in the active site are excluded, the correlation improves further (*R* = 0.62, *p* = 0.02; S6 Fig). These results show that our approach can reproduce experimental trends with sufficient accuracy.

The effectiveness of our approach is also demonstrated when comparing it to the study by Reetz and coworkers [15] applying iterative saturation mutagenesis to *Bs*LipA. The largest increase in *T*_50_ they have found for a variant containing a single point mutation in the first step was 4.3°C; our largest increase of 6.6°C compares favorably to this value. Four more steps of optimization and screening of about 8000 colonies then yielded two variants carrying five and seven mutations that showed an increase of *T*_50_ by 45°C. The study of Reetz *et al*. also differs from ours in a fundamental aspect: in the former study, those residues that showed the highest crystallographic B-factors, i.e., were the most mobile, were chosen as weak spots. In our study, weak spots constitute residues that segregate from large rigid, i.e. internally immobile, clusters during thermal unfolding.

In summary, these results suggest that our approach is a valuable, orthogonal complement to existing methods for rational protein design aimed at improving thermostability. The more thermostable variants can serve as starting points for further engineering of substrate scope and/or enantioselectivity by directed evolution, exploiting that enhanced thermostability promotes the ease of evolvability [75].

## Materials and Methods

### Constraint Network Analysis: Thermostability prediction

Constraint Network Analysis (CNA) predicts rigid and flexible regions within a biomolecule, which allows linking these static characteristics to the molecule’s stability and function [17,21]. CNA has been described in detail in refs. [17,21,35,76]. The approach has been used previously to predict the (thermodynamic) thermostability of proteins and to identify weak spot residues that, when mutated, are likely to improve thermostability [16,18,19].

In CNA, a protein is modeled as a *body-and-bar* network of bodies (atoms) and bars (covalent and noncovalent interactions). Each atom has six degrees of freedom, and each bar removes one degree of freedom [22]. An interaction between two atoms can be modeled as any number of bars between one and six depending on the strength of the interaction. Here, single covalent bonds (double and peptide bonds) were modeled as five (six) bars, hydrogen bonds and salt bridges (together referred to as “hydrogen bonds”) as five bars, and hydrophobic interactions as two bars. For hydrogen bonds a hydrogen bond energy *E*_HB_ is computed by a modified version of the potential by Mayo and coworkers [77] as described in ref. [26]. By successively removing noncovalent constraints from a network, a thermal unfolding of the protein is simulated [16,18,19,26]. Hydrogen bonds are removed from the network in increasing order of their strength [77], i.e., hydrogen bonds with an energy *E*_HB_ > *E*_cut_(σ) are discarded from the network of state σ. In the present study, *E*_cut_ values ranging from −0.1 kcal mol^−1^ to −6.0 kcal mol^−1^ with a step size of 0.1 kcal mol^−1^ were used. *E*_cut_ can be converted to a temperature using a linear relation introduced by Radestock and Gohlke [16,18], according to which the range of *E*_cut_ used in this study is equivalent to increasing the temperature of the system from 302 K to 420 K with a step size of 2 K. The rigidity of each network state σ during the thermal unfolding simulation is analyzed by the pebble game algorithm [23,24] as implemented in the FIRST program [25]. From these analyses, the change in the global rigidity characteristics is monitored by the cluster configuration entropy *H*_type2_ [76]. Finally, a phase transition temperature *T*_p_ is identified as the temperature when a largely rigid network becomes largely flexible. We showed that *T*_p_ can be used for predicting the thermodynamic thermostability of and identifying structural weak spots in a protein [16,18,19]. Usually, multiple phase transitions occur during the thermal unfolding of a protein because of its modular architecture, i.e., secondary structure elements can segregate from the largest rigid cluster as a whole [18].

### Constraint Network Analysis: Local rigidity indices

In contrast to global indices, local indices monitor rigidity at a residue level. One such index, the rigidity index *r*_i_, is defined for each covalent bond *i* between two atoms as the *E*_cut_ value during the thermal unfolding simulation at which the bond changes from rigid to flexible [76]. For a C_α_ atom-based representation, the average of the two *r*_i_ values of the two backbone bonds is taken. As a two-dimensional itemization of *r*_i_, a stability map *rc*_*ij*_ indicates for all residue pairs the *E*_cut_ value at which a rigid contact between the two residues *i*, *j* is lost, i.e., when the two residues stop belonging to the same rigid cluster [76]. From *rc*_*ij*_, a rigid cluster decomposition, i.e., a set of rigid clusters and flexible links in between, can be computed for each network state σ during the thermal unfolding simulation.

When the stability map *rc*_*ij*_ is filtered such that only rigid contacts between residues that are at most 5 Å apart from each other (measured as the distance between the closest atom pair of the two residues) are considered, a neighbor stability map results. This map helps focusing on short-range rigid contacts that can be directly modulated by mutagenesis with the aim to stabilize them for improving the overall stability of a protein.

In this study we use neighbor stability maps to analyze the (local) effect of mutations on the stability of rigid contacts of neighboring residues (S4 Fig). The increase in the strength of rigid contacts is calculated as the average over differences in *rc*_*ij*_ of the variant *versus* WT for all neighboring residue pairs (lower triangles in S4 Fig). The increase in the strength is measured in K.

### Generation of a structural ensemble of wild-type *Bs*LipA

Rigidity analyses using CNA are sensitive with respect to the input structure [45,78]. One way to improve the robustness is to carry out CNA on a structural ensemble derived from molecular dynamics (MD) simulations; then results (*T*_p_ values and stability maps) are averaged [19]. In the present study, MD simulations of WT *Bs*LipA were performed using the GPU accelerated version of PMEMD [79] of the AMBER 11 suite of programs [80,81] together with the ff99SB force field [82]. The X-ray crystal structure of *Bs*LipA with the highest resolution (PDB ID: 1ISP; resolution 1.3 Å) was used as input structure [83]. Hydrogen atoms were added using the REDUCE program [84] during which side-chains of Asn, Gln, and His were flipped if necessary to optimize the hydrogen bond network. Then, the system, neutralized by addition of sodium counter-ions, was solvated by a truncated octahedral box of TIP3P [85] water such that a layer of water molecules of at least 11 Å width covers the protein surface. The particle mesh Ewald method [86] was used with a direct-space non-bonded cutoff of 8 Å. Bond lengths involving hydrogen atoms were constrained using the SHAKE algorithm [87], and the time step for the simulation was 2 fs. After equilibration, a production run of unrestrained MD in the canonical ensemble (NVT) was performed to generate a trajectory of 100 ns length, with conformations extracted every 40 ps from the last 80 ns resulting in a structural ensemble of 2000 conformations. The ensemble was used to predict weak spot residues on *Bs*LipA.

### Strategy for predicting thermostabilzing mutations on *Bs*LipA

According to our strategy (Fig 1), the structural ensemble of 2000 conformations of WT *Bs*LipA (Fig 1a) was initially submitted to CNA for weak spot identification and prioritization. An average stability map was generated from individual stability maps for each conformation in the ensemble. A thermal unfolding trajectory showing average rigid cluster decompositions during the thermal unfolding simulation was reconstructed from the average stability map (Figs 1b and S7). For this we exploited that rigid cluster decompositions can be reconstructed from stability maps as the latter store *E*_cut_ (or temperature, according to the above mentioned linear relation) values for all residue pairs at which these residues cease to be in one rigid cluster during the thermal unfolding. The thermal unfolding trajectory was visually inspected for identifying transitions at which the rigidity of WT *Bs*LipA is substantially reduced using VisualCNA [88]. The inspection was done with a view that rigidifying contacts between the largest rigid cluster and residues that segregate at these substantial phase transitions should improve the thermostability of the protein by delaying the disintegration of the largest rigid cluster. Accordingly, at every transition, residues that are in the neighborhood of, and whose side-chains point towards the largest rigid cluster from which they segregated, were identified as potential weak spots (Fig 1c). Weak spot residues that showed a high sequence conservation (≥ 80% identity) in a multiple sequence alignment of 296 lipase class 2 sequences obtained from the Pfam database [89] were not considered further (Fig 1d).

Next, for modeling single-point site-saturation mutations, structures of all possible mutations at each weak spot residue were generated by the SCWRL program [50] using WT *Bs*LipA (PDB ID: 1ISP) as a template (Fig 1e). Conformations of side-chains of all residues within 8 Å of a mutated residue were re-predicted in order to allow for a local structural relaxation. The goodness of fit of the mutated side-chain in its environment was assessed using the ANOLEA server [51,52]. A variant was discarded if its average ANOLEA energy of the neighboring residues (≤ 5 Å of the mutation) is higher than the average energy of the same residues in WT by ≥ 2 kcal mol^-1^. For all variant structures, hydrogen atoms were added using REDUCE [84] in an identical way as done for WT *Bs*LipA (see section “Generation of a structural ensemble of WT *Bs*LipA” for details). The structures were minimized by 2000 steps of conjugate gradient minimization (including an initial steepest descent minimization for 100 steps) or until the root mean-square gradient of the energy was < 1.0*10^-4^ kcal mol^-1^ Å^-1^. The energy minimization was carried out with Amber11 [80] using the ff99SB force field [82] and the GB^OBC^ generalized Born model [90].

Finally, the generated variant structures were subjected to thermostability prediction and prioritization. In order to circumvent compute-intensive MD simulations for generating structural ensembles of each of the *Bs*LipA variants, the more efficient ENT^FNC^ approach [35] was used in connection with thermal unfolding simulations by CNA [21]. Ensembles of 1000 network topologies of all single point variants of *Bs*LipA were analyzed; for consistency, the WT *Bs*LipA structure was treated in the same way (including an energy minimization as described above). For each variant and WT, *T*_p_ was identified as the inflection point of the sigmoid with the larger change in *H*_type2_ using a double sigmoid function [19] fitted to *H*_type2_ *vs*. *T* curves. That way, in most cases, a late transition involving the final decay of the largest rigid cluster is identified as *T*_p_ [18] except when a very large loss of rigidity occurs during an early transition. Based on ensemble-averaged *T*_p_ (Fig 1f), variants were selected for experimental characterization of their thermostability and Michaelis-Menten kinetics. See S1 Text for details. Table 4 summarizes the required computing times.

## Supporting Information

S1 Fig Map of Δ*T*_p_ = *T*_p_ (variant) − *T*_p_ (wt) values for each mutation (abscissa) at each weak spot residue (ordinate) identified by CNA in WT *Bs*LipA. Weak spot residues are grouped by the major transition at which they are identified (Table 1 and Fig 2 in the main text). Mutations are colored according to thermostabilizing (red) or thermodestabilizing (blue) effects: Of the 239 single point mutations at the 13 weak spots identified from early transitions at low temperatures (T1 and T2), only four resulted in a higher *T*_p_ than WT *Bs*LipA; two of these increases were statistically significant (*p* < 0.05 according to Welch’s t-test [91]). At five weak spots identified at transition T3, seven mutations resulted in higher *T*_p_ values than WT *Bs*LipA; three of these increases were statistically significant. The most pronounced predicted thermostabilization both in terms of the number of variants showing increased *T*_p_ values (55, of which 27 were significant) and the magnitude of the *T*_p_ increase was observed for mutations at the nine weak spots identified at transition T4. Finally, nine mutations at four weak spot residues identified at the last transition T5 resulted in an increase in *T*_p_ compared to WT *Bs*LipA; six of these increases were significant. Weak spot residues that are highly conserved in the multiple sequence alignment of the lipase family (see the main text) are shown in gray. Mutations that led to energetically unfavorable structures as calculated by the ANOLEA server are shown as white stripes on gray color. Experimentally tested variants are marked by a black box.
(TIF)

S2 Fig Specific activities of *Bs*LipA variants between temperatures 40 and 60°C. The *Bs*LipA variants and the *p*NPP substrate solutions were incubated for 5 min at the indicated temperatures, and then the activity was measured at these temperatures. Variants G104I and I87W were inactive at these temperatures.
(TIF)

S3 Fig Specific activities of *Bs*LipA variants between temperatures 40 and 60°C. The *Bs*LipA variants and the *p*NPP substrate solutions were incubated for 30 min at the indicated temperatures, and then the activity was measured at these temperatures. Variants G104I, G104L, and I87W were inactive at these temperatures.
(TIF)

S4 Fig Differences in the stability of rigid contacts between WT and variants of *Bs*LipA: V54H (a), F58I (b), V96S (b). In the map, a red (blue) color indicates that a rigid contact in the variant is more (less) stable than in the WT (see color scale). The upper triangle shows differences in the stability values for all residue pairs; the lower triangle shows differences in the stability values only for residue pairs that are within 5 Å of each other, with values for all other residue pairs colored grey. Secondary structure elements as computed by the DSSP program [92,93] are indicated on both abscissa and ordinate and are labeled: α-helix (red rectangle), β-strand (green rectangle), loop (black line). Mutated residues are indicated by arrows. Blow-ups for secondary structure pairs of F58I described in the main text are shown.
(TIF)

S5 Fig Blow-ups of Fig 4d in the main text showing differences in the stability of “rigid contacts” between variant F58I and WT depicted on the variant structure: αB/loop β4-αB (a); αA/β strands 3,4,5 (b); αB/αC (c); loop αB-β5/loop αC-β6 (d); loop αC-β6/loop αD-β7 (e). Two residues form a “rigid contact” if they belong to one rigid cluster. A red (blue) stick connecting C_α_ atoms of two residues indicates that a rigid contact in the variant is more (less) stable than in the WT (see color scale). Only those contacts of variant F58I that are stabilized or destabilized by ≥ 2 K are shown for clarity; for the same reason, contacts between two residues of the same secondary structure element are not shown. Note that the blow-ups shown here are related to the blow-ups shown in S4b Fig.
(TIF)

S6 Fig Scatter plot of predicted Δ*T*_p_ values for all variants except the eight small-to-large residue mutations and the two mutations in the active site versus the experimental change in thermostability. (TIF)

S7 Fig Construction of average rigid cluster decompositions from using a structural ensemble. The input structure (1) is subjected to MD simulations for generating a structural ensemble (2). Stability maps are generated for each conformation in the ensemble using CNA (3). From all individual maps, an average stability map is generated (4). A trajectory showing the average rigid cluster decomposition during the thermal unfolding is then reconstructed from the average stability map (5).
(TIF)

S8 Fig SDS-PAGE of all variants and the WT that were purified using a N-terminal his-tag and the Ni-NTA purification method. After purification the samples were desalted and stored in 10 mM glycine buffer pH 11. The variant I87W was in all biological replicates not expressed properly and could only be purified in small amounts.
(TIF)

S1 Table *Bs*LipA variants with negative Δ*T*_p_ characterized experimentally. (PDF)

S2 Table Confusion matrix for two possible outcomes Δ*T* > 0 and Δ*T* < 0 for classifying 22 (15) *Bs*LipA variants with respect to predicted and experimental changes in thermostability related to WT *Bs*LipA. (PDF)

S3 Table *Bs*LipA variants and mutagenesis primer sequences. (PDF)

S1 Text Experimental characterization of thermostability and Michaelis-Menten kinetics. (PDF)

S2 Text Mann–Whitney U test. (PDF)
