# Supplementary figures and images for: Application of Rigidity Theory to the Thermostabilization of Lipase A from Bacillus subtilis
Source: PLoS Comput Biol. 2016 Mar 22;12(3):e1004754. doi: 10.1371/journal.pcbi.1004754 (PMC4803202; doi:10.1371/journal.pcbi.1004754)

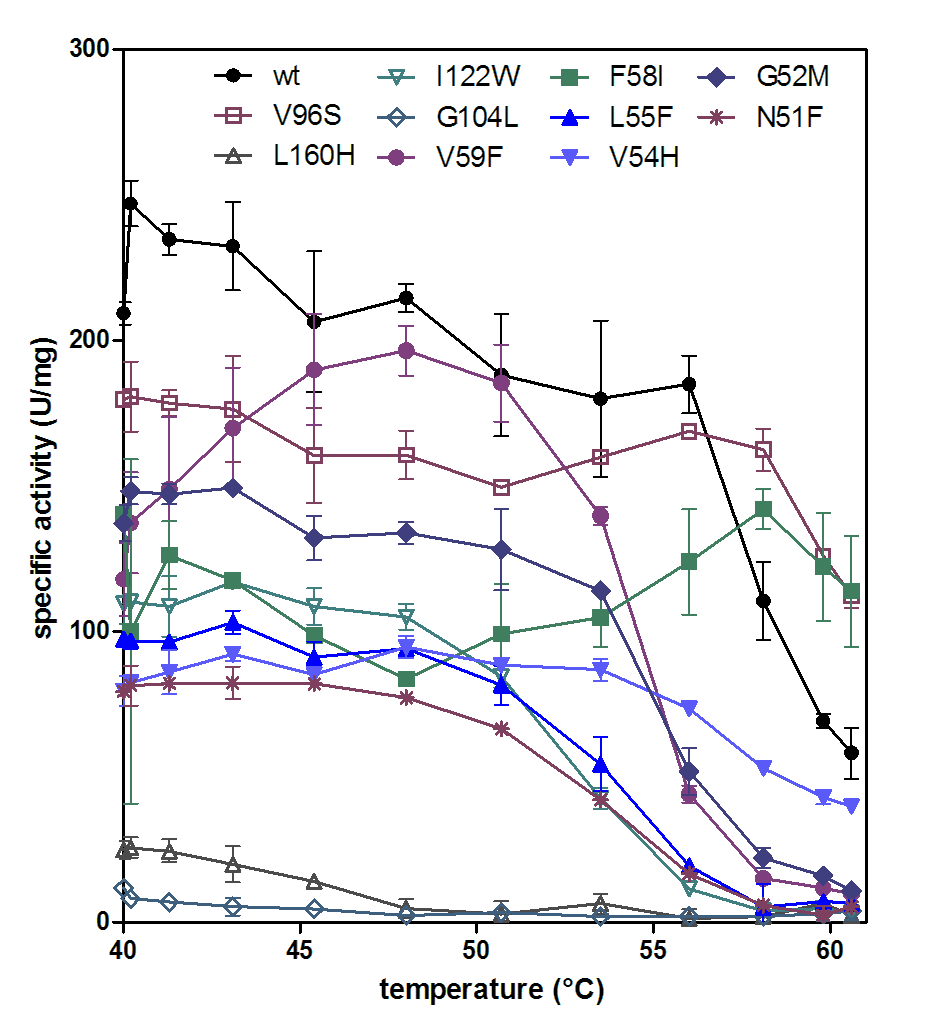

Supplement: S2 Fig — The BsLipA variants and the pNPP substrate solutions were incubated for 5 min at the indicated temperatures, and then the activity was measured at these temperatures. Variants G104I and I87W were inactive at these temperatures. (TIF) [file pcbi.1004754.s002.tif]

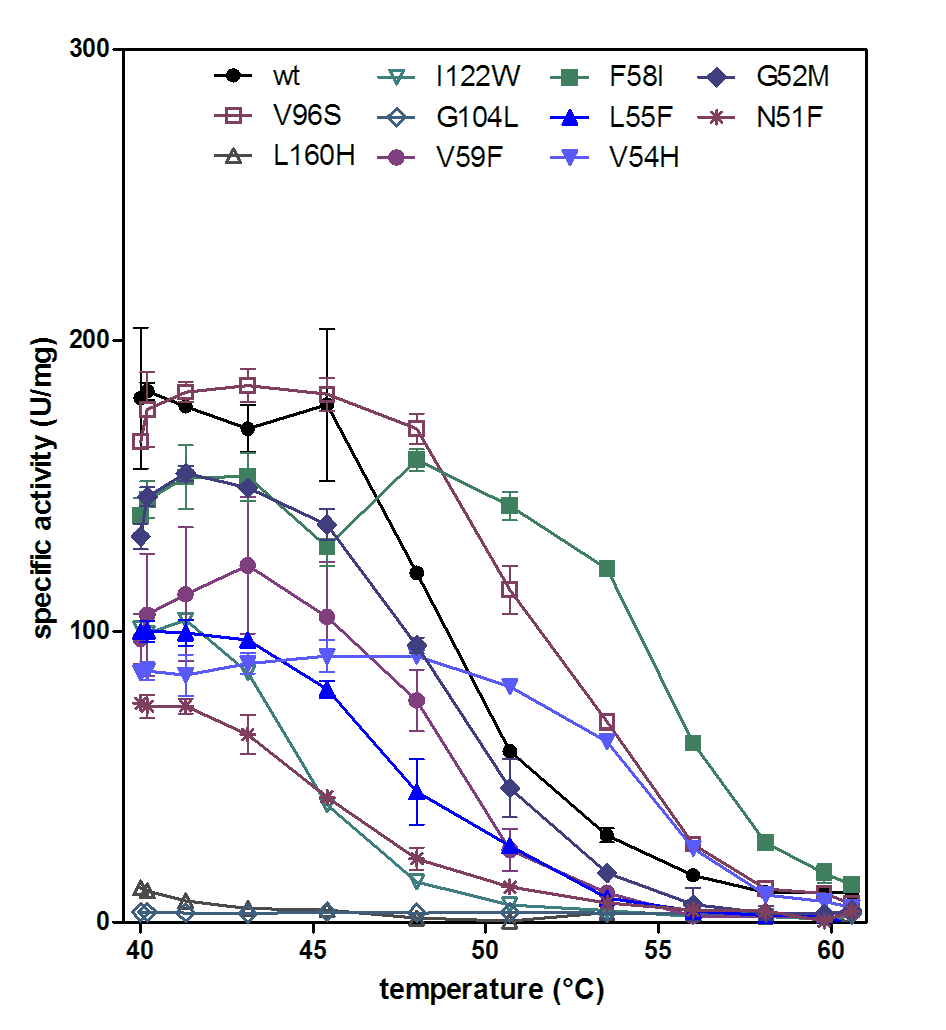

Supplement: S3 Fig — The BsLipA variants and the pNPP substrate solutions were incubated for 30 min at the indicated temperatures, and then the activity was measured at these temperatures. Variants G104I, G104L, and I87W were inactive at these temperatures. (TIF) [file pcbi.1004754.s003.tif]

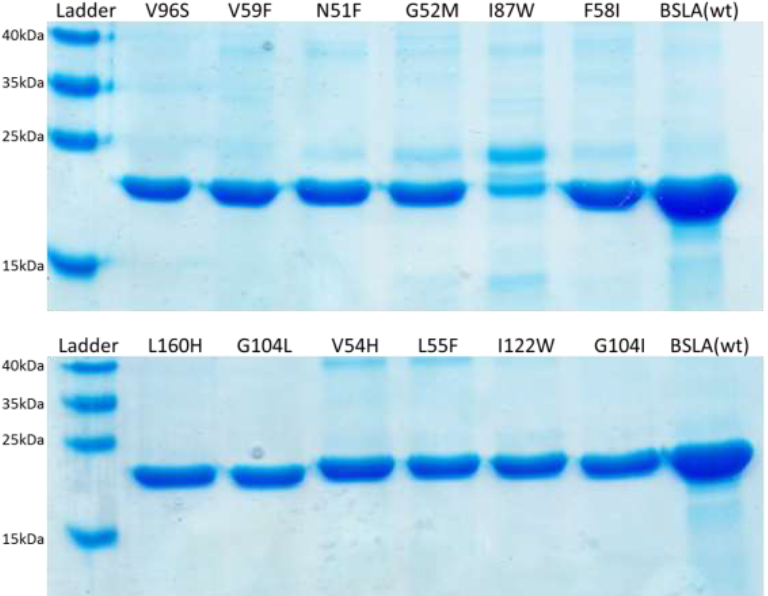

Supplement: S8 Fig — After purification the samples were desalted and stored in 10 mM glycine buffer pH 11. The variant I87W was in all biological replicates not expressed properly and could only be purified in small amounts. (TIF) [file pcbi.1004754.s008.tif]
