# Supplementary material for: Application of Rigidity Theory to the Thermostabilization of Lipase A from Bacillus subtilis
Source: PLoS Comput Biol. 2016 Mar 22;12(3):e1004754. doi: 10.1371/journal.pcbi.1004754 (PMC4803202; doi:10.1371/journal.pcbi.1004754)
Supplement: S1 Table — (PDF) [file pcbi.1004754.s009.pdf]

Table S1. *Bs*LipA variants with negative ΔT_p characterized experimentally.

***Bs*LipA variant**[a]	**Location of the mutation on secondary structure element**	**Phase transition of weak spot identification**	**Predicted ΔT_p**[b]	***T''*****$_{50}$**[c]
Wild-type	-	-	-	48.32
**L55V**	αB	T4	-9.45	44.04
**N48R**	αB	T4	-8.89	41.03
**N51P**	αB	T4	-7.73	42.88
**A105T**	αD	T3	-6.20	46.50
T83K[e]	αC	T5	-5.85	49.71
**V165A**	αF	T2	-4.37	45.87
**G46D**[e]	Loop β4 - αB	-[d]	-8.77	43.48
**G116K**[e]	Loop β6 - αD	-[d]	-8.61	42.85
**S56H**	Loop αD - β7	-[d]	-8.02	45.24
**D43G**	Loop β4 - αB	-[d]	-7.79	43.10

[a] Variants highlighted in bold show a significant decrease in T''_{50} compared to WT.

[b] Difference phase transition temperatures T_p (variant) − T_p (WT); in °C.

[c] The temperature at which the fraction of the activity to the initial activity (at RT) is 50% after incubating for 20 min and cooling to room temperature; in °C.

[d] The mutation was predicted at a non-weak spot.

[e] These mutations are considered "small-to-large" mutations.
