# Supplementary material for: Application of Rigidity Theory to the Thermostabilization of Lipase A from Bacillus subtilis
Source: PLoS Comput Biol. 2016 Mar 22;12(3):e1004754. doi: 10.1371/journal.pcbi.1004754 (PMC4803202; doi:10.1371/journal.pcbi.1004754)
Supplement: S2 Table — (PDF) [file pcbi.1004754.s010.pdf]

Table S2. Confusion matrix for two possible outcomes $\Delta T > 0$ and $\Delta T < 0$ for classifying 22 (15) *Bs*LipA variants with respect to predicted and experimental changes in thermostability related to WT *Bs*LipA.[a]

		**Experimental**	
		**$\Delta T > 0$**	**$\Delta T < 0$**
**Predicted**	**$\Delta T > 0$**	5[b] (3)	7[c] (2)
	**$\Delta T < 0$**	1 (0)	9 (7)

[a] See Table 2 and S1 Table for the raw data. No. of true positives (TP): 5 (3), no. of false positives (FP): 7 (2), no. of false negatives (FN): 1 (0), no. of true negatives (TN): 9 (7).

Sensitivity = TP / (exp. $\Delta T > 0$) = 5/6 = 83% (3/3 = 100%)

Specificity = TN / (exp. $\Delta T < 0$) = 9/16 = 56% (7/9 = 77%)

Accuracy = (TP + TN) / (Total population) = (5+9)/22 = 63% ((3+7)/12 = 83%).

Precision = TP / (pred. $\Delta T > 0$) = 5/(5+7) = 42% (3/(3+2) = 60%)

Random precision = (exp. $\Delta T > 0$) / (Total population) = (5+1)/22 = 27% ((3+0)/12 = 25%)

Gain in precision = precision / (random precision) = 1.6 (2.4)

Values in brackets result if the small-to-large residue mutations and the two mutations in the active site are excluded.

[b] Variants G52M und V59F were classified as experimental $\Delta T > 0$.

[c] Variants I87W, G104I, and G104L for which $\Delta T > 0$ was predicted but for which no activity could be measured (Table 2) were counted as false positives.
