# Supplementary material for: Application of Rigidity Theory to the Thermostabilization of Lipase A from Bacillus subtilis
Source: PLoS Comput Biol. 2016 Mar 22;12(3):e1004754. doi: 10.1371/journal.pcbi.1004754 (PMC4803202; doi:10.1371/journal.pcbi.1004754)
Supplement: S3 Table — (PDF) [file pcbi.1004754.s011.pdf]

Table S3. *Bs*LipA variants and mutagenesis primer sequences

Mutation	Forward primer sequence	Reverse Primer sequence
G104I	GTCGTGACGCTTGGCatcGCGAACCGTTTGACG	CGTCAAACGGTTCGCgatGCCAAGCGTCACGAC
G104L	GTCGTGACGCTTGGCctgGCGAACCGTTTGACG	CGTCAAACGGTTCGCcagGCCAAGCGTCACGAC
L55F	AACAATGGACCGGTAttcTCACGATTTGTGCAA	TTGCACAAATCGTGAgaaTACCGGTCCATTGTT
V59F	GTATTATCACGATTTttcCAAAAGGTTTTAGAT	ATCTAAAACCTTTTGgaaAAATCGTGATAATAC
I122W	CAGATCCAAATCAAtggATTTTATACACATCC	GGATGTGTATAAAATccaTTGATTTGGATCTG
L160H	GGACACATCGGCCTTcatTACAGCAGCCAAGTC	GACTTGGCTGCTGTAatgAAGGCCGATGTGTCC
N51F	GGCACAAATTATAACttcGGACCGGTATTATCA	TGATAATACCGGTCCgaaGTTATAATTTGTGCC
G52M	CACAAATTATAACAATatgCCGGTATTATCACGA	TCGTGATAATACCGGcatATTGTTATAATTTGTG
V54H	TATAACAATGGACCGcatTTATCACGATTTGTG	CACAAATCGTGATAAatgCGGTCCATTGTTATA
F58I	CCGGTATTATCACGAatcGTGCAAAAGGTTTTA	TAAAACCTTTTGCACgatTCGTGATAATACCGG
I87W	GAACACACTTTACTACtggAAAAATCTGGACGGC	GCCGTCCAGATTTTTccaGTAGTAAAGTGTGTTC
V96S	GACGGCGGAAATAAAagcGCAAACGTCGTGACG	CGTCACGACGTTTGCgctTTTATTTCCGCCGTC
