# Supplementary material for: Application of Rigidity Theory to the Thermostabilization of Lipase A from Bacillus subtilis
Source: PLoS Comput Biol. 2016 Mar 22;12(3):e1004754. doi: 10.1371/journal.pcbi.1004754 (PMC4803202; doi:10.1371/journal.pcbi.1004754)
Supplement: S1 Text — (PDF) [file pcbi.1004754.s012.pdf]

Supplemental materials and methods

Experimental characterization of thermostability and Michaelis-Menten kinetics

Materials

Phusion high fidelity polymerase, dNTPs, and the BCA protein quantification kit were obtained from Thermo Scientific (St. Leon Rot, Germany). Plasmid isolation and PCR cleanup was performed using the purification kits from Analytik Jena (Jena, Germany). Sequencing and synthesis of oligonucleotides was carried out by MWG eurofins (Ebersberg, Germany). The substrates for activity assays (*p*-nitrophenyl-palmitate (*p*NPP), *p*-nitrophenyl-decanoate (*p*NPD)) were purchased from Sigma Aldrich (Hamburg, Germany). Ni-NTA superflow resin and disposable PD-10 desalting columns were purchased from Macherey-Nagel (Düren, Germany).

Site directed mutagenesis

The *lipA* gene from *B. subtilis* encoding lipase without its signal sequence was cloned into pET22b+ (Novagen) with an N-terminal hexahistidine-tag fusion for purification. The site directed mutations (Table S3) were introduced with a modified Quikchange® protocol [1]. The amplification was carried out in two separate 25 µl reactions with each 10-50 ng of template, 0.2 pM of either the forward or reverse primer (Table S3), 0.2 mM dNTPs, 3% DMSO (v/v), and 1 U of Phusion polymerase in Phusion GC-buffer containing 7.5 mM $MgCl_2$. PCR conditions as follows: initial denaturation at 98°C for 10 min followed by 23 cycles of 98°C for 1 min, 55°C for 1 min, and 68°C for 3.5 min followed by final elongation at 68°C for 7 min. The PCR was paused after 5 cycles to combine the forward and reverse primer reaction and continued for the remaining 18 cycles. Template DNA was removed with 30U *Dpn*I at 37°C for 16 h. The hydrolysis reaction was stopped at 75°C for 20 min followed by a PCR clean up and eluted into 10 µl ddH_2O. An aliquot of 1 µl was transformed into *E. coli* DH5α electrocompetent cells and plated on selective LB plates. The plasmid DNA of the positive transformants was isolated and sequenced to ensure the successful site directed mutagenesis. Plasmid DNA with the desired mutation in *lipA* was stored at -20°C.

Cultivation and protein purification

The wild-type enzyme LipA and twelve variants were expressed in *E. coli* BL21(DE3) from a T7 promoter. An aliquot of 50 µl chemically competent *E. coli* BL21(DE3) was transformed

with 1 µl of plasmid DNA, and cells were grown overnight in 10 ml selective LB media. This preculture was used to inoculate the main expression culture in 250 ml of TB autodinducing media in a 5 l shaking flask, shaken at 150 rpm for 3 h at 37°C followed by 72 h at 15°C. Cells were collected by centrifugation for 45 min at 5000 rpm and lysed by passing three times through a French pressure cell at 500 bar (lysis buffer: 50 mM NaH_2PO_4, 300 mM NaCl adjusted to pH 8). The soluble fraction was incubated 30 min under mild agitation with 1 ml Ni-NTA Superflow Resin (Qiagen). The resin was then washed with 50 ml of 20 mM imidazole on a gravity flow column after which the protein was eluted with 250 mM imidazole into fractions of 1.5 ml each until there was no absorption detectable at 280 nm. The fractions with the highest absorption at 280 nm were applied to a disposable PD-10 desalting column according to manufacturer's description to remove imidazole. The protein concentration of the desalted sample was measured using the Micro BCA protein assay reagent (Thermo scientific), with bovine serum albumin in concentrations of 25 – 2000 µg/ml as a standard [2]. The sample purity was analyzed by SDS-PAGE.

Thermostability assay: T'_{50} values

The protein concentration was adjusted to 0.3 mg/ml in 10 mM glycine buffer pH 11 as determined by a BCA measurement. The enzyme stock was diluted 10-fold into 50 µl of 50 mM NaP_i/KP_i pH 7 and incubated at different temperatures between 40°C to 60°C in a PCR cycler for 30 min. The lipase activity was measured with a *p*NPP substrate solution [3] (1.6 mM *p*NPP, 10% isopropanol, 50 mM NaP_i/KP_i pH 7, 1 mg/ml gum arabic, 2 mg/ml sodium desoxycholate) warmed up in a separate PCR cycler 5 min prior to the measurement. For the measurement 50 µl of substrate solution were added to 50 µl of the sample, and the hydrolysis rate was quantified by the change in absorption at 410 nm for the duration of 5 min in a SpectraMax Plus plate reader (Molecular Devices, Sunnyvale, CA). T'_{50} values were obtained from this as the temperature at which the fraction of the activity to the initial activity (at 40ºC) is 50% after incubating for 30 min with a measurement taken at the incubation temperature.

Michaelis-Menten kinetics

The initial rates of hydrolysis as a function of the substrate concentration were measured by following the change in absorption at 410 nm for 60 s with 10 s lag time in 1 ml disposable cuvettes using a SpectraMax Plus plate reader with a built in cuvette port (Molecular Devices, Sunnyvale, CA). The substrate *p*NPP is thermostable and was thus chosen for the

thermostability assays; however, its low solubility at higher concentrations did not allow determining kinetic parameters. Therefore, kinetic constants were determined using the substrate *p*NPD, which is less stable but soluble also at higher concentrations. The measurement was started by mixing 990 µl of a 40°C substrate solution (6 – 1600 µM *p*NPD in 10% isopropanol, 50 mM NaPi/KPi pH 7, 1 mg/ml gum arabic, 2 mg/ml sodium desoxycholate) with 10 µl of room temperature enzyme solution (0.05 mg/ml in 50 mM NaPi/KPi pH 7, 1 mg/ml gum arabic, 2 mg/ml sodium desoxycholate). The K_M and k_{cat} values for each variant were derived by nonlinear fitting of the Michaelis–Menten curve using the software Graphpad PRISM (GraphPad Software, Inc., San Diego, CA) and the protein concentration from a BCA measurement as described earlier.

Thermostability assay for negative controls: T''_{50} values

To determine the thermostability of the negative controls (Table S1), culture supernatants of *E. coli* BL21 cells that release *Bs*LipA into the media were obtained as described previously [4]. The *Bs*LipA supernatant (diluted in 50 mM sodium phosphate buffer, pH 7.2) was incubated at temperatures from 40 to 60°C for 20 min, cooled at 4°C for 20 min, and equilibrated at room temperature for 20 min. Enzymatic activity was assayed using *p*NPP substrate solution and plotted against the incubation temperature to calculate the T''_{50}. For the WT *Bs*LipA and five of its variants (N51F, L55F, F58I, V59F, V96S; Table 2), a significant ($p < 0.0001$) and very good (Pearson correlation $R = 0.987$) correlation between T''_{50} values determined here and T'_{50} values determined above for the purified variants was obtained.

References

1. Edelheit O, Hanukoglu A, Hanukoglu I (2009) Simple and efficient site-directed mutagenesis using two single-primer reactions in parallel to generate mutants for protein structure-function studies. BMC Biotechnology 9: 61.
2. Smith PK, Krohn RI, Hermanson GT, Mallia AK, Gartner FH, et al. (1985) Measurement of protein using bicinchoninic acid. Analytical Biochemistry 150: 76-85.
3. Winkler UK, Stuckmann M (1979) Glycogen, hyaluronate, and some other polysaccharides greatly enhance the formation of exolipase by Serratia marcescens. Journal of Bacteriology 138: 663-670.
4. Fulton A, Frauenkron-Machedjou VJ, Skoczinski P, Wilhelm S, Zhu L, et al. (2015) Exploring the protein stability landscape: Bacillus subtilis lipase A as a model for detergent tolerance. Chembiochem 16: 930-936.
