# Supplementary material for: Application of Rigidity Theory to the Thermostabilization of Lipase A from Bacillus subtilis
Source: PLoS Comput Biol. 2016 Mar 22;12(3):e1004754. doi: 10.1371/journal.pcbi.1004754 (PMC4803202; doi:10.1371/journal.pcbi.1004754)
Supplement: S2 Text — (PDF) [file pcbi.1004754.s013.pdf]

Supplemental results

Mann–Whitney *U* test

We applied the Mann–Whitney *U* test (also called the Wilcoxon rank-sum test) [1] as implemented in *R* [2] as a nonparametric test of the null hypothesis that the two samples of *Bs*LipA variants in Table 2 in the main text (which were *predicted* to have a higher thermostability, "sample 1") as well as in Table S1 (which were *predicted* to have a lower thermostability, "sample 2") come from the same population in terms of the *experimental* relative thermostability with respect to WT *Bs*LipA. The null hypothesis is tested against the alternative hypothesis that the probability of finding a *Bs*LipA variant with higher experimental relative thermostability in sample 1 than in sample 2 is greater than the probability of finding a *Bs*LipA variant with higher experimental relative thermostability in sample 2 than in sample 1 (one-sided test).

Considering all *Bs*LipA variants in Table 2 and Table S1 except I87W, G104I, and G104L, for which no activity could be measured and, thus, for which no ordinal number can be specified, the median values for sample 1 and sample 2 of experimental relative thermostabilities are 0.34 and -4.56°C; Mann–Whitney $U = 71$, n1 = 9, n2 = 10, $p = 0.017$ one-sided.

Considering all *Bs*LipA variants in Table 2 and Table S1 except small-to-large residue mutations and the two mutations in the active site, the median values for sample 1 and sample 2 of experimental relative thermostabilities are 3.55 and -4.28°C; Mann–Whitney $U = 35$, n1 = 5, n2 = 7, $p = 0.001$ one-sided.

Hence, for a significance level of 0.05, in both cases the null hypothesis is rejected in favor of the alternative hypothesis. Phrased differently, an increase in thermostability predicted by our approach significantly points to increased experimental thermostability.

References

1. Mann HB, Whitney DR (1947) On a Test of Whether one of Two Random Variables is Stochastically Larger than the Other. Ann Math Stat 18: 50-60.
2. Ihaka R, Gentleman R (1996) R: R: A Language for Data Analysis and Graphics. J Comput Graph Stat 5: 299-314.
